# Relapse-free survival in a pediatric patient with recurrent *EZH2*-mutant melanoma treated with adjuvant tazemetostat

**DOI:** 10.1038/s41698-025-00826-8

**Published:** 2025-02-21

**Authors:** Erin E. Resch, Stavriani C. Makri, Paola Ghanem, Ezra G. Baraban, Kenneth J. Cohen, Alan R. Cohen, Evan J. Lipson, Christine A. Pratilas

**Affiliations:** 1 Division of Pediatric Oncology, Department of Oncology, The Sidney Kimmel Comprehensive Cancer Center, Johns Hopkins University School of Medicine, Baltimore, MD USA; 2 Department of Medicine, Johns Hopkins University School of Medicine, Baltimore, MD USA; 3 Department of Pathology, Johns Hopkins University School of Medicine, Baltimore, MD USA; 4 Division of Pediatric Neurosurgery, Department of Neurosurgery, Johns Hopkins University School of Medicine, Baltimore, MD USA; 5 Department of Oncology, Bloomberg~Kimmel Institute for Cancer Immunotherapy and The Sidney Kimmel Comprehensive Cancer Center, Johns Hopkins University School of Medicine, Baltimore, MD USA

**Keywords:** Cancer, Molecular medicine, Oncology

## Abstract

Enhancer of zeste homolog 2 (EZH2) is an essential epigenetic regulator of H3K27 histone methylation and is mutated or overexpressed in a wide variety of cancers. In melanoma, EZH2 overexpression contributes to excessive trimethylation of H3K27 on tumor suppressor genes and has been proposed to be a mechanism of tumor progression and metastasis. EZH2-targeted therapies have been successfully used to treat patients with follicular lymphoma and epithelioid sarcoma, but their clinical use in melanoma has not been described. Here, we describe a pediatric patient with multiply relapsed melanoma harboring an *EZH2* A692V missense mutation, treated adjuvantly with the EZH2 inhibitor tazemetostat, who experienced a prolonged relapse-free survival.

## Introduction

Enhancer of zeste homolog 2 (EZH2) functions as a catalytic subunit of the polycomb repressor complex 2 (PRC2) and thereby facilitates trimethylation of histone H3 at lysine 27 (H3K27me3) to promote transcriptional repression^1^. EZH2 mutations have emerged as important therapeutic targets in cancer, with the EZH2 inhibitor tazemetostat gaining FDA approval in 2020 for the treatment of patients with advanced epithelioid sarcoma and follicular lymphoma^2–4^. Next-generation sequencing has identified EZH2 overexpression and/or alterations across several cancer types—including melanoma—and is associated with aggressive disease^5–7^. In melanoma preclinical studies, EZH2 overexpression has been implicated in the escape of immune surveillance, silencing of tumor suppressors and apoptosis pathways, and shaping the interactions between melanoma cells and the microenvironment^8–11^. Although an objective response rate of 69% was observed in the *EZH2-* mutant cohort of a phase 2 trial in patients with follicular lymphoma, EZH2 inhibitors have so far shown limited effectiveness in most solid tumors, particularly in the pediatric population^1,4,12^. Clinical trials have not yet demonstrated the efficacy of EZH2 inhibition in patients with *EZH2*-mutant melanoma. Here, we describe a pediatric patient with multiply relapsed melanoma harboring an *EZH2* mutation, who achieved a prolonged relapse-free survival following adjuvant treatment with tazemetostat.

## Results

### Case presentation

**Fig. 1:**
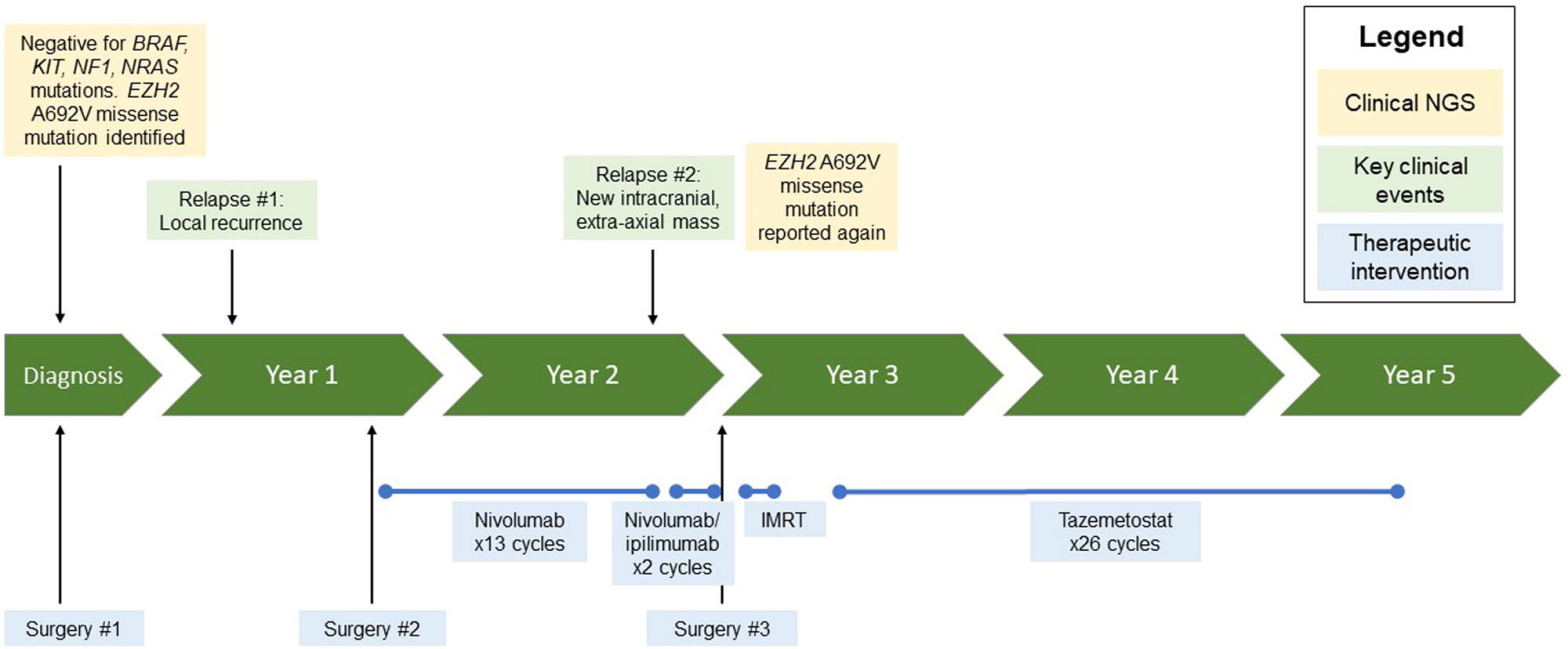
Case report timeline. Timeline summarizing the patient’s clinical history, highlighting key genomic findings and treatment interventions. *IMRT* intensity modulated radiation therapy, *NGS* next generation sequencing.

**Fig. 2:**
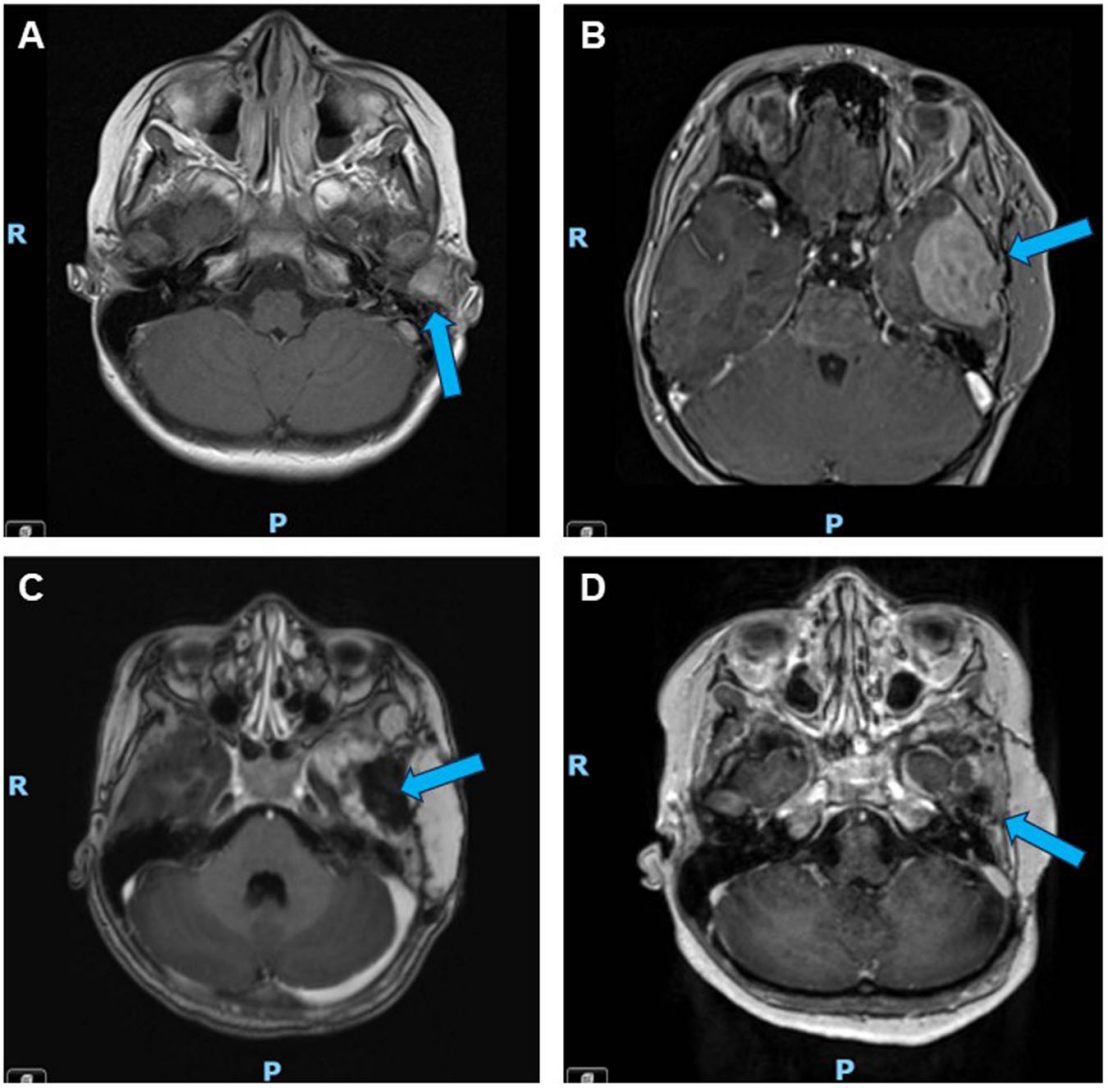
Axial T1 MRI images of the brain through the course of treatment. **A** First relapse; a 1.4 cm tumor was identified in the left external auditory canal three months after initial debulking surgery and prior to any systemic treatment. **B** Second relapse; after one year of adjuvant nivolumab, the patient experienced a second relapse when a new 4.0 cm mass was identified along the floor of the left middle cranial fossa with mass effect on the left temporal lobe. **C** Post-operative imaging; the patient underwent intracranial tumor resection after two cycles of neoadjuvant ipilimumab/ nivolumab. **D** Representative surveillance imaging while on tazemetostat; eighteen months post-operatively and fifteen months into tazemetostat therapy, the patient continued to demonstrate no evidence of relapsed disease. Blue arrows indicate the location of the tumor or resection site.

A 10-year-old female with no significant past medical history was referred to our institution from abroad for the management of relapsed melanoma. Ten months prior to presentation, she had developed left ear pain, discharge, and hearing loss and was evaluated at a local hospital where she was noted to have an “abscess-like lesion” around the left external auditory canal (Fig. 1). Despite initial treatment with drainage and antibiotics, her symptoms persisted, and the lesion grew. Computed tomography (CT) scan demonstrated a mass causing complete opacification of the left external auditory canal. She underwent debulking surgery two months after her initial presentation and pathology confirmed a diagnosis of melanoma, *BRAF* wild-type. Whole body ^18^F-fluorodeoxyglucose positron emission tomography (FDG-PET) and CT chest, abdomen, and pelvis scans showed uptake only in an ipsilateral cervical lymph node; fine needle aspiration (FNA) of this node was negative for melanoma. Three months post-operatively, the patient developed bleeding and a bulging mass from the left external auditory canal. Magnetic resonance imaging (MRI) revealed a locally recurrent tumor obstructing the auditory canal (Fig. 2A). She was referred to our institution for further management of relapsed disease.

On presentation to our institution (eight months after her original diagnosis and four months from the first recurrence), she had an approximately 3 cm, round, ulcerated mass protruding posterior to the left inferior pinna, with purulent drainage from the left auditory canal. CT and FDG-PET evaluations at our institution confirmed a 4.4 cm mass arising from the left external auditory canal with hypermetabolic enlarged ipsilateral level 2a and 2b cervical lymph nodes. Next-generation sequencing (NGS) was performed on the original tumor (block obtained from her home institution) and was negative for *BRAF, KIT, NF1*, and *NRAS* mutations, with homozygous loss of exon 3 of *PTEN* (chr10q), whole arm loss of chr6q, and detection of multiple coding variants, including both known pathogenic variants and variants of unknown significance (VUS) (Table 1). She underwent left auricular circumferential wide local excision, left total auriculectomy, and neck dissection. Pathologic examination of the primary mass, surrounding soft tissue, and 42 regional lymph nodes confirmed the recurrence of melanoma involving the dermis, subcutaneous tissue, and bone, without angiolymphatic invasion; all lymph nodes and surrounding tissue margins were negative for disease. She received adjuvant therapy with nivolumab for one year, according to melanoma standard of care recommendations, without significant adverse events^13^.

**Table 1:** Next-generation sequencing (NGS) comparing first and second relapse

Chr:Pos	Base change	Reference database ID	Gene	AA change	VAF (%) (first relapse)	VAF (%) (second relapse)
chr16:11012343	G > A	rs552040932	*CIITA*	p.E1037K	48.79	46.87
chr3:134967315	C > T	rs904442404	*EPHB1*	p.P885L	48.22	47.9
chr7:148506437	G > A	COSM220529	*EZH2*	p.A692V	26.68	40.81
chr16:89831370	G > C	rs587778315; COSM2923648	*FANCA*	p.D902E	48.1	49.43
chr1:150551774	C > T		*MCL1*	p.R78Q	48.58	49.48
chr7:82451900	T > C	rs149360770	*PCLO*	p.K4901R	49.56	56.39
chr7:82578986	A > G		*PCLO*	p.F3640L	51.21	50.22
chr7:103294611	T > C	rs150850005	*RELN*	p.I495V	46.77	46.88
chr6:152708224	T > A		*SYNE1*	p.I2824L	12.74	14.86
**Additional mutations**			**First relapse**		**Second relapse**	
Homozygous loss, exon 3, *PTEN* (chr10q)			Present		Not reported	
Whole arm loss, chr6q			Present		Not reported	

*AA* amino acid, *VAF* variant allele frequency.

NGS comparing changes in the patient's tumor mutations at the first relapse (prior to nivolumab treatment) and at the second relapse (after thirteen cycles of nivolumab and two cycles of ipilimumab/nivolumab). No new mutations were identified between the two NGS studies.

MRI at the completion of one year of nivolumab demonstrated a second relapse (Fig. 2B), with a 4.0 cm extra-axial mass along the floor of the left middle cranial fossa with associated enhancement of the adjacent dura, bony erosion of the adjacent left sphenoid and temporal bone, and extension into the left temporomandibular joint. She received two cycles of neoadjuvant ipilimumab and nivolumab and the tumor demonstrated interval growth to 5.2 cm. She underwent an intracranial tumor resection after these two cycles (Fig. 2C). While interval growth may have suggested the possibility of pseudoprogression, pathologic examination of this tumor confirmed relapsed melanoma with a minimal degree of inflammation, demonstrating sheets of viable tumor and relatively sparse inflammatory infiltrates that were not prominent. NGS of this tumor specimen was again negative for *BRAF, KIT, NF1*, and *NRAS* mutations, but was again notable for a missense mutation in *EZH2* (c.2075 C > T, p.A692V) (Table 1). Immunochemistry for H3K27me3 demonstrated retained (normal) protein expression in tumor cells.

Post-operatively, the intent had been to administer adjuvant ipilimumab/nivolumab. However, the patient developed markedly elevated liver function tests (AST, ALT) consistent with grade 4 hepatitis, presumed secondary to immune checkpoint inhibitors (ICIs). She was managed with a prolonged steroid taper and no further adjuvant ipilimumab/nivolumab was administered. She was treated with four weeks of intensity-modulated radiation therapy (IMRT 62.5 Gy to high-risk areas, 50 Gy to intermediate-risk areas). Germline testing for melanoma-associated cancer predisposition genes (Invitae Melanoma Panel) was negative for mutations in *BAP1, BRCA1/2, CDK4, CDKN2A, MC1R, MITF, POT1, PTEN, RB1, TERT*, and *TP53*.

The patient’s extremely elevated risk of future recurrence prompted the need for adjuvant systemic treatment. Further immunotherapy was contraindicated given her ongoing steroid treatment for grade 4 hepatitis. Cytotoxic chemotherapy has poor success in relapsed melanoma and targeted therapies are generally preferred, but no traditionally targetable alterations, such as *BRAF* or *KIT*, were identified^13^. After discussion at the Johns Hopkins Molecular Tumor Board and careful consideration, she was started on the EZH2 inhibitor tazemetostat based upon the presence of the pathogenic *EZH2*^*A692V*^ mutation identified on NGS^14^. Tazemetostat was begun three months after completion of radiation therapy and administered at an oral dose of 1200 mg/m^2^/dose twice daily, the recommended phase II dose (RP2D) established in a pediatric phase I study of tazemetostat in rhabdoid tumors and INI1-negative tumors and used in Children’s Oncology Group NCI-COG Pediatric MATCH Consortium protocol APEC1621C for CNS-involved tumors^12,15^. She completed 24 months (26 cycles) of tazemetostat and interval imaging throughout treatment showed no evidence of disease recurrence (representative imaging after fifteen months of tazemetostat shown in Fig. 2D). The patient tolerated tazemetostat well, without requiring dose reductions or holds for adverse events. Reported adverse events during treatment were occasional nausea and occasional mild, bilateral lower extremity myalgias that were managed conservatively. She remained relapse-free for fourteen months after the completion of this therapy.

Following the submission of the original draft of this manuscript, we learned that local intracranial recurrence was again detected on routine surveillance MRI imaging. In total, the patient therefore experienced a relapse-free interval of three years and seven months from the last resection and radiation, which included interval treatment with tazemetostat of two years’ duration.

## Discussion

We describe a patient with multiply recurrent melanoma harboring an *EZH2* A692V missense mutation—a pathogenic variant most commonly described in follicular lymphoma—who experienced a prolonged relapse-free survival with adjuvant tazemetostat. EZH2 is overexpressed or mutated in many cancer types, including melanoma, but the clinical success of EZH2-targeted therapies to date has been limited to epithelioid sarcoma and follicular lymphoma. To our knowledge, our patient is the first described with *EZH2*^A692V^-mutant melanoma treated with tazemetostat as precision-targeted therapy.

EZH2 functions as the catalytic subunit of the histone methyltransferase polycomb repressive complex 2 (PRC2), that catalyzes the trimethylation of lysine 27 of histone 3 (H3K27me3). Trimethylation of H3K27 is an epigenetic regulator that canonically promotes chromatin compaction and transcriptional repression. In cancer, silencing of tumor suppressor genes through H3K27 trimethylation is associated with aggressive disease and has been implicated as a mechanism for oncogenesis and metastasis^5–8,16,17^. *EZH2* mutations have been described in numerous cancer types including melanoma, hematologic malignancies, breast cancer, prostate cancer, liver and biliary cancers, lung cancer, and endometrial cancer, while *EZH2*^A692V^ hotspot mutations have been most commonly described in follicular lymphoma and diffuse large B cell lymphoma^18–25^.

Tazemetostat is an epigenetic therapy and first-in-class EZH2 inhibitor that gained FDA accelerated approval in 2020 for patients greater than or equal to 16 years old with *SMARCB1*/INI1-deficient metastatic or locally advanced epithelioid sarcoma (ES) and adults with *EZH2*-mutated relapsed or refractory follicular lymphoma (FL). In their respective phase II trials, patients with ES achieved a 15% objective response rate (ORR), while patients with *EZH2*-mutated FL had a 69% ORR^3,4,12^. A pediatric phase 1 study was conducted in children with relapsed or refractory malignant rhabdoid tumor, atypical teratoid rhabdoid tumor, other *SMARCB1*-deficient tumors, or synovial sarcoma and saw responses in 14% of tumors in the dose expansion cohort population, with a 24% response rate in ATRT^15^. The National Cancer Institute-Children’s Oncology Group Pediatric Molecular Analysis for Therapy Choice (MATCH) trial also investigated the efficacy of tazemetostat for *SMARCB1*/INI1-deficient or *EZH2*-mutated refractory solid tumors, brain tumors, lymphoma, or histiocytic disorders in a biomarker selected population, including those with loss of function of *SMARCA1* or *SMARCA4*, or hotspot *EZH2* mutations. While the MATCH trial was successful in demonstrating the feasibility of enrolling in a group-wide, histology-agnostic screening protocol using molecular biomarker selection and the agent was overall well tolerated, the primary efficacy endpoint was not reached, with only one objective response (ORR 1/20 or 5%)^12^. Four additional patients achieved stable disease and remained in the study for at least nine months (range 9–26 months). Three patients (including two with Ewing sarcoma and one with ependymoma) enrolled based on *EZH2* mutations experienced tumor progression and discontinued treatment after two cycles of tazemetostat. No melanoma patients were treated in any of these pediatric trials. In these studies, some patients achieved prolonged stable disease, with 67% of ES responses and 25% of MATCH tumor responses demonstrating stability for more than six months.

While the most common somatic mutations in melanoma occur in the RAF-MEK-ERK pathway (*BRAF, NRAS, NF1, MAP2K1, KIT*), *EZH2* mutations have been identified in up to 6–7% of melanoma cases^26–28^. *EZH2* activating mutations or copy number alterations in melanoma result in transcriptional silencing of tumor suppressor genes, immune response genes, and differentiation factors, contributing to poorer overall survival and metastasis-free survival^7,8,25,26,29,30^. Recently, EZH2 overactivation by the nucleosome remodeling enzyme Mi-2β was identified as a mechanism of immune evasion in melanoma^11^. No clinical trials have yet reported the efficacy of tazemetostat in patients with melanoma, but preclinical studies have suggested EZH2-targeted therapies may interfere with melanoma progression and metastasis and could be a target for combinations with either ICI or BRAF-targeted therapy^26^.

In the case of our patient, following the second intracranial recurrence, tazemetostat was selected given the drug’s success in follicular lymphoma, wherein *EZH2*^A692V^ hotspot mutations represent a recurrent, actionable alteration and were included in the clinical trial that led to its FDA approval^4,18,19^. While patients with localized melanoma have an excellent 5-year overall survival (OS) of >99% following surgical resection, those with relapsed or distant metastatic disease have a 5-year OS of less than 35% and require systemic treatment. Conventional chemotherapy is often poorly effective, but the response to systemic treatment has improved significantly in the last two decades with the use of ICIs and BRAF-selective inhibitors (BRAFi)^31–38^. In our patient’s case, progression had occurred following two lines of therapy with ICIs, and no mutations predictive of response to BRAF/MEK-targeted therapy were identified. Additionally, the patient’s ongoing steroid taper to treat grade 4 hepatitis secondary to nivolumab/ipilimumab precluded the use of further ICIs or intensive conventional chemotherapy regimens. With very limited options, tazemetostat represented a therapeutic intervention unsupported by clinical experience, but the near certainty of future intracranial recurrence was a compelling argument to pursue adjuvant targeted therapy.

An absolute interpretation of this patient’s response to tazemetostat was limited by several factors: first, there was no measurable disease at the start of treatment; second, her tumor recurrence was also treated with two cycles of neoadjuvant ipilimumab/nivolumab and adjuvant radiation. We felt the neoadjuvant ipilimumab/nivolumab was less likely to have induced a tumor response, as the tumor demonstrated an approximate 30% increase in size after these two cycles of treatment without evidence of prominent inflammatory infiltrate to suggest pseudoprogression. Additionally, although radiation is not consistently effective in treating melanoma, the patient did receive a high dose of adjuvant radiation that confounds the true treatment effect attributable to adjuvant tazemetostat. Despite these limitations in assessing the degree of disease response that is attributable to tazemetostat, we nevertheless find this patient’s case compelling: despite experiencing two relapses and progression through immunotherapy in the two years since her diagnosis, with precision-targeted therapy with tazemetostat, the patient experienced a relapse-free period of over three-and-a-half years.

This report highlights the potential clinical application of EZH2 inhibitors in melanoma, particularly for patients with *EZH2*^A692V^ hotspot mutations. Although tazemetostat monotherapy yields poor ORR in clinical trials for solid tumors, up to 25–67% of patients demonstrate disease stability ≥6 months, supporting a role for tazemetostat in *EZH2*-mutated solid tumors in combination with other therapies or in the maintenance setting to prevent disease relapse. Further studies are needed to determine which *EZH2* or *SMARCB1* alterations are most predictive of response to EZH2-targeted therapies and the best administration sequence of these drugs in *EZH2*-mutated melanomas.

## Methods

### Case report

A single pediatric patient treated at Johns Hopkins Hospital (JHH) was identified for this case analysis. A retrospective chart review was conducted to extract clinical data relevant to this report. The patient’s parent provided written informed consent to publish this anonymized data.

### Next generation sequencing

Molecular testing was conducted by the Clinical Laboratory Improvement Amendments (CLIA)-certified Johns Hopkins Medical Laboratory at the Johns Hopkins Hospital (Baltimore, MD). Targeted NGS was performed on formalin-fixed, paraffin-embedded tissue sections with the Johns Hopkins Solid Tumor Panel, version 6 (https://pathology.jhu.edu/jhml-services/test-directory/ngs-solid-tumor-panel).

## Data Availability

No datasets were generated or analysed during the current study.
